# Supplementary material for: Definition and surgical timing in cauda equina syndrome–An updated systematic review
Source: PLoS One. 2023 May 4;18(5):e0285006. doi: 10.1371/journal.pone.0285006 (PMC10159340; doi:10.1371/journal.pone.0285006)
Supplement: S1 File — (DOCX) [file pone.0285006.s002.docx]

**EMBASE Search strategy**

| Search # | Search term | Results |
| --- | --- | --- |
| 1 | cauda equina syndrome.af. |  |
| 2 | (case report or abstract).af. |  |
| 3 | animal.af. |  |
| 4 | (cauda equina syndrome not (case report or abstract)).af. |  |
| 5 | (cauda equina syndrome not (case report or abstract) not animal).af. |  |
| 6 | limit 5 to english language [Limit not valid in Your Journals@Ovid; records were retained] |  |
| 8 | Limit 9 to yr=”1990 –Current” | 376 |

**MEDLINE Search strategy**

Ovid Medline

| **Search #** | **Search term** | **Results** |
| --- | --- | --- |
| 1 | exp cauda equina syndrome/ |  |
| 2 | (case report or abstract).mp. [mp=title, abstract, original title, name of substance word, subject heading word, keyword heading word, protocol supplementary concept word, rare disease supplementary concept word, unique identifier, synonyms] |  |
| 3 | Animals/ |  |
| 4 | 1 not 2 |  |
| 5 | 4 not 3 |  |
| 6 | limit 5 to english language |  |
| 7 | limit 6 to yr=”1990 -Current” | 87 |

**CINAHL Plus Search strategy**

| **Search #** | **Search term** | **Search Options** | **Results** |
| --- | --- | --- | --- |
| 1 | Cauda equina syndrome |  |  |
| 2 | Cauda equina syndrome NOT (case report or abstract) |  |  |
| 3 | Cauda equina syndrome NOT (case report or abstract) NOT (animal) |  |  |
| 4 | Cauda equina syndrome NOT (case report or abstract) NOT (animal) | **Narrow by Language:**- english |  |
| 5 | Cauda equina syndrome NOT (case report or abstract) NOT (animal) | **Narrow by Language:**- english  **Limiters** - Publication Year: 1990-201 | 227 |

**List of all included studies**

| **No.** | **Title** | **Year** | **Journal** |
| --- | --- | --- | --- |
| (1) | Urgent discectomy: Clinical features and neurological outcome | 2016 | Surgical Neurology International |
| (2) | The Cauda Scale – Validation for Clinical Practice | 2020 | British Journal of Neurosurgery |
| (3) | Factors Associated with Recovery in Motor Strength, Walking Ability, and Bowel and Bladder Function after Traumatic Cauda Equina Injury | 2021 | Journal of NeuroTrauma |
| (4) | Impact of timing on surgical outcome in patients with cauda equina syndrome caused by lumbar disc herniation | 2016 | Medicinski Glasnik |
| (5) | Time to Surgery and Outcomes in Cauda Equina Syndrome: An Analysis of 45 Cases | 2016 | World Neurosurgery |
| (6) | Percutaneous Endoscopic Lumbar Discectomy as an Emergent Surgery for Cauda Equina Syndrome Caused by Lumbar Disc Herniation | 2020 | Pain Physician |
| (7) | Obesity and spinal epidural lipomatosis in cauda equina syndrome | 2018 | The Spine Journal |
| (8) | Cauda equina syndrome due to disk herniation: Long-term functional prognosis | 2019 | Neurocirugia |
| (9) | Cauda equina syndrome secondary to lumbar disc herniation: Surgical delay and its relationship with prognosis | 2016 | Revista Española de CirugíaOrtopédica y Traumatología |
| (10) | Evaluation of nationwide referral pathways, investigation and treatment of suspected cauda equina syndrome in the United Kingdom | 2019 | British Journal of Neurosurgery |
| (11) | Is scan-negative cauda equina syndrome a functional neurological disorder? A pilot study | 2020 | European Journal of Neurology |
| (12) | Limited sequence MRI to improve standards of care for suspected cauda equina syndrome | 2020 | The Bone and Joint Journal |
| (13) | Influence of timing of surgery on Cauda equina syndrome: Outcomes at a national spinal centre | 2018 | Journal of Orthopaedics |
| (14) | A service evaluation of patients suspected of Cauda Equina Syndrome referred to accident and emergency departments from a national telephone triage service | 2020 | Musculoskeletal Science and Practice |
| (15) | Scan-Negative Cauda Equina Syndrome | 2021 | Neurology |
| (16) | The clinical features and outcome of scan-negative and scan-positive cases in suspected cauda equina syndrome: a retrospective study of 276 patients | 2018 | Journal of Neurology |
| (17) | Timing of Surgical Decompression for Cauda Equina Syndrome | 2019 | World Neurosurgery |
| (18) | Time to implement a national referral pathway for suspected cauda equina syndrome: review and outcome of 250 referrals | 2018 | British Journal of Neurosurgery |
| (19) | Factors affecting urinary outcome after delayed decompression in complete cauda equina syndrome: "A regression model study" | 2021 | European Journal of Trauma and Emergency Surgery |
| (20) | A prospective study of the role of bladder scanning and post-void residual volume measurement in improving diagnostic accuracy of cauda equina syndrome | 2020 | The Bone & Joint Journal |
| (21) | Early surgery determines recovery of motor deficits in lumbar disc herniations-a prospective single-center study | 2021 | Acta Neurochirurgica |
| (22) | Do we know the outcome predictors for cauda equine syndrome (CES)? A retrospective, single-center analysis of 60 patients with CES with a suggestion for a new score to measure severity of symptoms | 2017 | European Spine Journal |
| (23) | Cauda Equina Syndrome: presentation, outcome, and predictors with focus on micturition, defecation, and sexual dysfunction | 2017 | European Spine Journal |
| (24) | Lumbar spinal canal MRI diameter is smaller in herniated disc cauda equina syndrome patients | 2017 | PLoS One |
| (25) | The long term outcome of micturition, defecation and sexual function after spinal surgery for cauda equina syndrome | 2017 | PLoS One |
| (26) | Cauda Equina Syndrome: A Review of 15 Patients Who Underwent Percutaneous Transforaminal Endoscopic Lumbar Discectomy (PTELD) Under Local Anaesthesia | 2020 | Malaysian Orthopaedic Journal |
| (27) | Treatment of cauda equina syndrome caused by lumbar disc herniation with percutaneous endoscopic lumbar discectomy | 2015 | Acta Neurologica Belgica |
| (28) | Are we neglecting sexual function assessment in suspected cauda equina syndrome? | 2019 | Surgeon |
| (29) | Elsberg syndrome- A rarely recognized cause of cauda equina syndrome and lower thoracic myelitis | 2017 | Neurology Neuroimmunology and Neuroinflammation |
| (30) | Long-term rates of bladder dysfunction after decompression in patients with cauda equina syndrome | 2021 | The Spine Journal |
| (31) | Examination of the predictive power of electromyography and urodynamic study in patients with Cauda equina syndrome (horse tail syndrome) | 2016 | Acta Informatica Medica |
| (32) | Out of hours magnetic resonance imaging for suspected cauda equina syndrome: lessons from a comparative study across two centres | 2021 | Ann R Coll Surg Engl |
| (33) | Does surgical decompression alleviate neglected cauda equina syndromes attributed to lumbar disc herniation and/or degenerative canal stenosis? | 2020 | Surgical Neurology International |
| (34) | Early intervention in cauda equina syndrome associated with better outcomes: a myth or reality? Insights from the Nationwide Inpatient Sample database (2005-2011) | 2017 | The Spine Journal |
| (35) | Urgent operation improves weakness in cauda equina syndrome due to lumbar disc herniation | 2019 | Turkish Journal of Physical Medicine and Rehabilitation |
| (36) | Bladder Scans and Postvoid Residual Volume Measurement Improve Diagnostic Accuracy of Cauda Equina Syndrome | 2019 | Spine |
| (37) | Suspected cauda equina syndrome: no reduction in investigation, referral and treatment during the COVID-19 pandemic | 2021 | Ann R Coll Surg Engl |
| (38) | Analysis of clinical and neurological outcomes in patients with cauda equina syndrome caused by acute lumbar disc herniation: A retrospective-prospective study | 2017 | Oncotarget |
| (39) | Full Endoscopic Lumbar Discectomy Versus Laminectomy for Cauda Equina Syndrome | 2021 | International Journal of Spine Surgery |
| (40) | Efficacy of delayed decompression of lumbar disk herniation causing cauda equina syndrome | 2014 | Orthopedics |
| (41) | Complications associated with surgical stabilization of high-grade sacral fracture dislocations with spino-pelvic instability | 2006 | Spine |
| (42) | Cauda equina syndrome caused by intervertebral lumbar disk prolapse: mid-term results of 22 patients and literature review | 2002 | Orthopedics |
| (43) | Delayed presentation of cauda equina syndrome secondary to lumbar disc herniation: functional outcomes and health-related quality of life. | 2001 | Canadian Journal of Emergency Medicine |
| (44) | Outcome of spinal decompression in cauda equina syndrome presenting late in developing countries: case series of 50 cases | 2011 | European Spine Journal |
| (45) | Predictive value of clinical characteristics in patients with suspected cauda equina syndrome | 2009 | European Journal of Neurology |
| (46) | Epidemiological study of cauda equina syndrome | 2013 | Acta Ortopedica Brasileira |
| (47) | Spinal instability secondary to metastatic cancer | 1991 | The Bone & Joint Journal |
| (48) | Cauda equina syndrome as a postoperative complication in five patients operated for lumbar disc herniation | 2001 | Spine |
| (49) | Cauda equina syndrome: outcome and implications for management | 2003 | British Journal of Neurosurgery |
| (50) | Cauda equina syndrome: factors affecting long-term functional and sphincteric outcome | 2007 | Spine |
| (51) | Predictors of outcome in cauda equina syndrome | 1999 | European Journal of Spine |
| (52) | Cauda equina syndrome: an audit. can we do better? | 2004 | Journal of Orthopaedic Medicine |
| (53) | Cauda equina -syndrome (CES) from lumbar disc herniations | 2009 | Clinical Spine Surgery |
| (54) | Cauda equina syndrome treated by surgical decompression: the influence of timing on surgical outcome | 2007 | European Spine Journal |
| (55) | Cauda Equina Syndrome secondary to lumbar disc herniation | 2008 | Acta Orthopaedica Belgica |
| (56) | Posterior epidural migration of herniated lumbar disc fragment Clinical article | 2011 | Journal of Neurosurgery: Spine |
| (57) | Cauda equina syndrome secondary to lumbar disc herniation | 1993 | Neurosurgery |
| (58) | Medical realities of cauda equina syndrome secondary to lumbar disc herniation | 2000 | Spine |
| (59) | Study on different surgical approaches for acute lumber disk protrusion combined with Cauda Equina Syndrome | 2014 | International Journal of Clinical and Experimental Pathology |
| (60) | Postoperative lumbar epidural hematoma: does size really matter? | 2008 | Spine |
| (61) | Does early surgical decompression in cauda equina syndrome improve bladder outcome? | 2015 | Spine |
| (62) | Primary Spinal Tumor Mortality Score (PSTMS): a novel scoring system for predicting poor survival | 2014 | The Spine Journal |
| (63) | Cauda equina syndrome: evaluation of the clinical outcome | 2014 | European Review for Medical and Pharmacological Sciences |
| (64) | Causes and outcomes of cauda equina syndrome in medico-legal practice: a single neurosurgical experience of 40 consecutive cases | 2011 | British Journal of Neurosurgery |
| (65) | Conus medullaris and cauda equina tumors: clinical presentation, prognosis, and outcome after surgical treatment. Clinical article | 2014 | Journal of Neurosurgery: Spine |
| (66) | Urodynamic evaluation of surgical outcome in patients with urinary retention due to central lumbar disc prolapse | 2003 | Neurourology and Urodynamics |
| (67) | Morbidity of early spine surgery in the multiply injured patient | 2014 | Orthopaedic Surgery |
| (68) | Transverse sacral fractures with anterior displacement | 2008 | European Spine Journal |
| (69) | Decompression and Lumbopelvic Fixation for Sacral Fracture - Dislocations with Spino-pelvic Dissociation | 2006 | Journal of Orthopaedic Trauma |
| (70) | The clinical study of repairing cauda equina fibres with fibrin glue after lumbar fracture and dislocation | 2010 | Spinal Cord |
| (71) | Lumbopelvic fixation for multiplanar sacral fractures with spinopelvic instability | 2012 | Injury |
| (72) | Is cauda equina syndrome being treated within the recommended time frame? | 2011 | Neurosurgery |
| (73) | Do the Spinal Pathologies that Accompany Lumbar Disc Disease Affect Surgical Prognosis? | 2006 | Turkish Neurosurgery |
| (74) | Clinical classification of cauda equina syndrome for proper treatment | 2010 | Acta Orthopaedica |
| (75) | Lumbar Disc Prolapse: Management and Outcome Analysis of 96 Surgically treated Patients | 2002 | Journal of the Pakistan Medical Association |
| (76) | Intra-Operative Vertebroplasty Combined with Posterior Cord Decompression | 2014 | International Neuroradiology |
| (77) | Displaced spinopelvic dissociation with sacral cauda equina syndrome: outcome of surgical decompression with a preliminary management algorithm | 2012 | European Spine Journal |
| (78) | The role of calcium deposition in the ligamentum flavum causing a cauda equina syndrome and lumbar radiculopathy | 1995 | Paraplegia |
| (79) | Factors predicting outcomes of mechanical sciatica: a review of 1092 cases | 2004 | Joint Bone Spine |
| (80) | Sequestered extrusion of lumbar disk: experimental model, clinical picture, diagnosis and treatment | 2003 | Acta Clinica Croatica |
| (81) | The Value of Interhospital Transfer and Emergency MRI for Suspected Cauda Equina Syndrome: A 2-year retrospective study | 2008 | Annals of The Royal College of Surgeons of England |
| (82) | Cauda Equina Syndrome Following Decompression for Spinal Stenosis | 2011 | Global Spine Journal |
| (83) | Cauda equina syndrome in ankylosing spondylitis: successful treatment with lumboperitoneal shunting | 2010 | Spine |
| (84) | Improved symptoms and lifestyle more than 20 years after untethering surgery for primary tethered cord syndrome | 2011 | Neurourology and Urodynamics |
| (85) | Does rectal examination have any value in the clinical diagnosis of cauda equina syndrome? | 2012 | British Journal of Neurosurgery |
| (86) | Surgical evaluation and management of lumbar synovial cysts: The Mayo Clinic Experience | 2000 | Journal of Neurosurgery |
| (87) | Comparative analysis of patients with cauda equina syndrome versus an unaffected population undergoing spinal surgery | 2014 | Spine |
| (88) | Rehabilitation Outcome of Individuals with Nontraumatic Myelopathy Resulting from Spinal Stenosis | 1998 | Journal of Spinal Cord Medicine |
| (89) | Intraspinal epidermoid tumor of the cauda equina region: seven cases and a review of the literature | 2012 | Clinical Spine Surgery |
| (90) | Analysis of Clinical and Surgical Outcomes of Upper Lumbar Disk Herniations | 2015 | Neurosurgery Quarterly |
| (91) | Cauda equina lesions as a complication of spinal surgery | 2010 | European Spine Journal |
| (92) | Outcomes in patients admitted for rehabilitation with spinal cord or cauda equina lesions following degenerative spinal stenosis | 2004 | Disability and Rehabilitation |
| (93) | Spinal Myxopapillary Ependymoma Neurological Deterioration in Patients Treated with Surgery | 2009 | Spine |
| (94) | Spondylolisthesis treated by a single-stage operation combining decompression with in situ posterolateral and anterior fusion. An analysis of eleven patients who had long-term follow-up | 1990 | The Journal of Bone & Joint Surgery |
| (95) | Indication for Partial Vertebral Osteotomy and Realignment in Posterior Spinal Fixation for Osteoporotic Thoracolumbar Vertebral Collapse with Neurological Deficits | 2016 | Neurologia medico-chirurgica |
| (96) | Midline disk herniations of the lumbar spine | 1993 | Southern Medical Journal |
| (97) | Is Cauda Equina Surgery Safe Out-of-Hours? A Single United Kingdom Institute Experience | 2022 | World Neurosurgery |
| (98) | The Effect of COVID-19 National Lockdown on the Time from Presentation to Surgery of Patients with Suspected Cauda Equina Syndrome: Two UK Tertiary Centres Study | 2022 | World Neurosurgery |
| (99) | Body mass index has an impact on preoperative symptoms but not clinical outcome in acute cauda equina syndrome | 2021 | Nature Scientific reports |
| (100) | Minimally Invasive Transformainal Lumbar Interbody Fusion (TLIF) Compared with Open TLIF for Acute Cauda Equina Syndrome: A retrospective Single-Centre Study with Long-Term Follow-Up | 2022 | World Neurosurgery |
| (101) | Evaluation of the role of anal tone and perianal sensation examination in the assessment of suspected cauda equina syndrome | 2021 | British Journal of Neurosurgery |
| (102) | Lumbar decompression surgery for cauda equina syndrome - comparison of complication rates between daytime and overnight operating | 2022 | Acta Neurochir (Wien) |
| (103) | Improvement in Neurogenic Bowel and Bladder Dysfunction Following Posterior Decompression Surgery for Cauda Equina Syndrome: A Prospective Cohort Study | 2021 | Neurospine |
| (104) | Do safety-net hospitals provide equitable care after decompressive surgery for acute cauda equina syndrome? | 2021 | Clinical Neurology and Neurosurgery |
| (105) | Demographics of Cauda Equina Syndrome: A population based incidence study | 2021 | Neuroepidemiology |
| (106) | Physical Examination Is Predictive of Cauda Equina Syndrome: MRI to Rule Out Diagnosis is unnecessary | 2022 | Global Spine Journal |
| (107) | Post-void ultrasound in suspected cauda equina syndrome - data from medicolegal cases and relevance to magnetic resonance imaging scanning | 2022 | International Orthopaedics  Neuroepidemiology |
| (108) | The morbidity of out-of-hours surgery for Cauda Equina Syndrome | 2022 | British Journal of Neurosurgery |
| (109) | Rates of Future Lumbar Fusion in Patients with Cauda Equina Syndrome Treated With Decompression | 2022 | Journal of the American Academy of Orthopaedic Surgeons... |
| (110) | Cauda Equina Syndrome Poor Recovery Prognosis Despite Early Treatment | 2022 | Spine |

Details regarding the time duration between CES symptoms or admission and definition of the symptoms.

| Paper | Timing from | Symptoms defined | Details of Timing |
| --- | --- | --- | --- |
| Aly et al 2014 [40] | Symptoms | D sphincter disturbance | 1 to 3 months after sphincter disturbance |
| Beculic et al 2016 [4] | Symptoms | ND | <2d, 2-5d, 5-10d, 10-30d, >30d |
| Bellabarba et al 2006 [41] | Symptoms | ND | M: 6days R(1-30d) |
| Buchner et al 2002 [42] | Symptoms | D onset of urinary dysfunction | M: 44hrs R(4hrs-7days) |
| Busse et al 2001 [43] | Symptoms | ND | M: 6.19days R(1.6-14.3d) |
| Dhatt et al 2011 [44] | Symptoms | D perianal anaesthesia and disturbances in micturition | M: 12.2 days R(1-35d) |
| Domen et al 2009 [45] | Symptoms | D urinary retention or other alarming symptoms | M: 5.8 days since autonomic symptoms and 24hrs from admission to hospital |
| Foruria et al 2016 [9] | Symptoms | D genitourinary symptoms | <48hrs, >48hrs |
| Fuso et al 2013 [46] | Symptoms | ND | M: 18 +/- 24 days R (5-115 days) |
| Galasko et al 1991 [47] | Symptoms | D complete paraplegia or urinary retention | <18hrs from urinary retention |
| Henriques et al 2001 [48] | Symptoms | D 2 patients- complete paraplegia, 3 patients- slight paraparesis, PR numbness, loss of tone, urinary incontinence | 24-36hrs or 36-48hrs |
| Hussain et al 2003 [49] | Symptoms | D PR sensory loss or urinary dysfunction | <24 hrs of admission to the unit, median of 1 day for urinary symptoms and 6 days for PR sensory loss |
| Kennedy et al 1999 [51] | Symptoms | ND | M: 14hrs, R(6-24hrs) for good outcome group. Mean 30hrs R(6-70hrs) poor outcome group |
| McCarthy et al, 2007 [50] | Symptoms | D sphincteric symptoms | < 24hrs, 24-48 hrs, > 48hrs |
| Ng et al 2004 [52] | Symptoms | ND | M: 58hrs (between symptom onset and GP contact) 128hrs (from GP to specialist referral) 67 hrs (from MRI to surgery) |
| Olivero et al 2009 [53] | Symptoms | D urinary incontinence/ retention and sacral numbness | <24 hrs, 24-48hrs, >48hrs, R (60hrs to 2 weeks) |
| Qureshi et al 2007 [54] | Symptoms | D autonomic | M: 131hrs R(6-627hrs) |
| Raj et al 2008 [55] | Symptoms | D urinary symptoms | Acute (27hrs- 6 days) Insidious (15d-3m) |
| Albert et al 2016 [1] | Symptoms | ND | <24hrs |
| Sengoz et al 2011 [56] | Symptoms | ND | M: 4.2days R(1-10d) |
| Shapiro et al 1993 [57] | Symptoms | D urinary symptoms | R (<24hrs to more than 30 days) |
| Shapiro et al 2000 [58] | Symptoms | D urinary symptoms | M: 12.5 hrs R (7-40) for 20 patients and M: 9 days delay for 24 other patients |
| Shen et al 2014 [59] | Symptoms | ND | within 48hrs of CES symptoms |
| Sokolowski et al 2008 [60] | Symptoms | D bilateral motor and sensory deficits with diminished rectal tone | R (2-5d of procedure) |
| Srikandarajah et al 2015 [61] | Symptoms | D urinary symptoms | <48hrs, <72hrs >72 hrs |
| Szoverfi et al 2014 [62] | Symptoms | ND | M: 8.7m R (0-253 months) |
| Tamburrelli et al 2014 [63] | Symptoms | ND | <24hrs, >24hrs, <36hrs, >48hrs |
| Todd et al 2011 [64] | Symptoms | D after Cauda Equina Syndrome with Retention (CESR) | CESR> 48hrs, CESR 24-48hrs, <24hrs after CESR |
| Wostrack et al 2014 [65] | Symptoms | ND | M: 24m R(4d-20yrs) |
| Yamanishi et al 2003 [66] | Symptoms | D urinary retention | M: 42 hrs |
| Bydon et al 2016 [5] | Symptoms | D At least 4 of: bladder dysfunction, saddle anaesthesia, lower extremity weakness, lower extremity sensory disturbance, bowel dysfunction, or acute lower back or leg pain | Symptom onset to surgery mean time 62 hours (7.8-348 hours) <12 hours, between 12-24hrs, between 24-48 hrs, and >48 hrs |
| Konig et al 2017 [22] | Symptoms | D Urinary retention & incontinence, rectal incontinence, genital hypoesthesia, perianal hypoesthesia, reduced rectal tone, partial saddle anesthesia, complete saddle anesthesia, (Berlin score- not validated) | Admitted to hospital more than 48h after onset of CES. 76% of cohort treated within the first 24h |
| Heyes et al 2018 [13] | Symptoms | D BASS classification | <24hrs, 24-48hrs, >48rsh |
| Delgado-Lopez et al 2019 [8] | Symptoms | D Gleave and McFarlane criteria | Median time 78hrs, R (12-720), from diagnosis to surgery median 24hrs R (5-120), 4 had surgery <48 hrs, 18 over 48 hrs |
| Uckun et al 2019 [35] | Symptoms | D Low-back pain, leg pain, muscle weakness, incontinence, and sphincter control | Time from admission to surgery M 7.6 +/-5.2 hours, Symptom start to surgery: 83.8±11.0 |
| Sath et al 2020 [33] | Symptoms | D CES-R bladder retention and incontinence, CES-I incomplete bladder involvement | Bladder Sx - 3 to 24 months. Pt w/ CES-I had longer duration before surgery (8.25months mean), CES-R was shorter (mean 5.5m) |
| Hoeritzauer et al 2021 [15] | Symptoms | D Fraser criteria | ND |
| Jha et al 2021 [19] | Symptoms | D Fraser criteria | Time to surgery defined as time to decompression from onset of bladder dysfunction, time treated as a continuous variable, all operated >72 hrs |
| Kogl et al 2021 [21] | Symptoms | D Vesicorectal dysfunction | Time variable dichotomised at <72h or over. 4 CES patients experienced symptoms <24h before emergency discectomy, 2 after 48hrs |
| Galvin et al 2014 [67] | Trauma |  | M: 0.8 days from injury |
| Sapkas et al 2008 [68] | Trauma |  | 0-15days after the injury |
| Schildhauer et al 2006 [69] | Trauma |  | M: 6 days R (1-30days) |
| Sun et al 2010 [70] | Trauma |  | M: 4.14d R(3-7d) |
| Tan et al 2012 [71] | Trauma |  | M: 9.5days R(2-42d) |
| Attabib et al 2021 [3] | Trauma |  | Median 26.5 hrs (IQR 53) 79=> 24h, 83 >24h |
| Arrigo et al 2011 [72] | Admission |  | <24hrs, 24-48hrs, >48hrs |
| Kotil et al 2006 [73] | Admission |  | R(24hrs to 10 days), 3 within 48hrs |
| Shi et al 2010 [74] | Admission |  | within 8hrs from CES diagnosis made by clinician |
| Korse et al 2017 [23] | Admission |  | 24h, 96h, 120h, 138h, 168h, 192h, 216h |
| Korse et al 2017 [24] | Admission |  | <24h, 24-48h, 48-72h, 96h, 120h, 138h, 216h |
| Korse et al 2017 [25] | Admission |  | 7-12h, 13-24h, 25-48h, 49-72h, >72h |
| Krishnan et al 2020 [26] | Admission |  | All patients received surgery within 24h of the presentation. However, only 4 patients presented within 24h of the onset of bladder dysfunction |
| Hogan et al 2019 [17] | Admission |  | 78.3% patients underwent intervention on day 0-1 (16,379/20,924) 21.7% underwent delayed intervention, day 2 or later (4545/20,924) |
| Hussain et al 2018 [18] | Referral |  | Urgent surgery defined as <48h from referral, 29 within 48 hrs, 3 after 48 hrs |
| Fountain et al 2019 [10] | Referral |  | ND |
| Chen et al 2020 [6] | Radiological confirmation |  | All patients were taken to surgery <24h. Mean time was 15 hours +/-5.5 hours |
| Woodfield et al 2021 [37] | Radiological confirmation |  |  |
| Mirza et al 2022 (97) | Symptoms | D Urinary symptoms, saddle anaesthesia, lower back pain, bowel dysfunction, lower limb weakness, lower limb sensory, sexual symptoms | Time to surgery divided into <48h or >48h. 83% operated within 48h |
| Baraka et al 2021 (98) | Hospital admission |  | Time calculated from hospital admission to surgery – 87% operated within 48h |
| Butenschoen et al 2021 (99) | Symptoms | D Urinary symptoms and sexual symptoms | Median symptom duration before surgery reported as 2 days, IQR 1-4 days |
| Seidel et al 2022 (109) | Hospital admission |  | Divided patients into 3 groups, <48h, 48h-10d, 10d-30d. 86% operated within 48h |
| Todd et al 2022 (107) | MRI scan |  | 85% of patients were operated within 24h |
| Woodfield et al 2022 (105) | Hospital admission |  | 95% of patients underwent surgical decompression the day of the admission |

Abbreviations: CES – I: Cauda equina syndrome – Incomplete, CES-R: Cauda equina syndrome – with retention, D: Defined, M: Mean, ND: Not defined, PR: Per rectal, R: Range.

**References**

1. Albert R, Lange M, Brawanski A, Schebesch K-M. Urgent discectomy: Clinical features and neurological outcome. Surg Neurol Int [Internet]. 2016;7:17. Available from: https://pubmed.ncbi.nlm.nih.gov/26958423

2. Angus M, Berg A, Carrasco R, Horner D, Leach J, Siddique I. The Cauda Scale - Validation for Clinical Practice. Br J Neurosurg. 2020/04/21. 2020;34(4):453–6.

3. Attabib N, Kurban D, Cheng CL, Rivers CS, Bailey CS, Christie S, et al. Factors Associated with Recovery in Motor Strength, Walking Ability, and Bowel and Bladder Function after Traumatic Cauda Equina Injury. J Neurotrauma. 2020/09/11. 2021;38(3):322–9.

4. Bečulić H, Skomorac R, Jusić A, Alić F, Imamović M, Mekić-Abazović A, et al. Impact of timing on surgical outcome in patients with cauda equina syndrome caused by lumbar disc herniation. Med Glas. 2016/07/28. 2016;13(2):136–41.

5. Bydon M, Lin JA, De la Garza-Ramos R, Macki M, Kosztowski T, Sciubba DM, et al. Time to Surgery and Outcomes in Cauda Equina Syndrome: An Analysis of 45 Cases. World Neurosurg. 2016/01/03. 2016;87:110–5.

6. Chen C, Fan P, Huang L, Zhen H, Liu L, Wang Y. Percutaneous Endoscopic Lumbar Discectomy as an Emergent Surgery for Cauda Equina Syndrome Caused by Lumbar Disc Herniation. Pain Physician. 2020/06/11. 2020;23(3):E259-e264.

7. Cushnie D, Urquhart JC, Gurr KR, Siddiqi F, Bailey CS. Obesity and spinal epidural lipomatosis in cauda equina syndrome. Spine J. 2017/08/02. 2018;18(3):407–13.

8. Delgado-López PD, Martín-Alonso J, Martín-Velasco V, Castilla-Díez JM, Galacho-Harriero A, Ortega-Cubero S, et al. Cauda equina syndrome due to disk herniation: Long-term functional prognosis. Neurocir (Astur Engl Ed). 2019/06/07. 2019;30(6):278–87.

9. Foruria X, Ruiz de Gopegui K, García-Sánchez I, Moreta J, Aguirre U, Martínez-de Los Mozos JL. Cauda equina syndrome secondary to lumbar disc herniation: Surgical delay and its relationship with prognosis. Rev Esp Cir Ortop Traumatol. 2016/03/08. 2016;60(3):153–9.

10. Fountain DM, Davies SCL, Woodfield J, Kamel M, Majewska P, Edlmann E, et al. Evaluation of nationwide referral pathways, investigation and treatment of suspected cauda equina syndrome in the United Kingdom. Br J Neurosurg. 2019/08/14. 2019;0(0):1–11.

11. Gibson LL, Harborow L, Nicholson T, Bell D, David AS. Is scan-negative cauda equina syndrome a functional neurological disorder? A pilot study. Eur J Neurol. 2020/02/20. 2020;27(7):1336–42.

12. Gnanasekaran R, Beresford-Cleary N, Aboelmagd T, Aboelmagd K, Rolton D, Hughes R, et al. Limited sequence MRI to improve standards of care for suspected cauda equina syndrome. Bone Jt J. 2020/04/02. 2020;102-b(4):501–5.

13. Heyes G, Jones M, Verzin E, McLorinan G, Darwish N, Eames N. Influence of timing of surgery on Cauda equina syndrome: Outcomes at a national spinal centre. J Orthop [Internet]. 2018;15(1):210–5. Available from: https://www.sciencedirect.com/science/article/pii/S0972978X17302672

14. Higginson R, Letheren A, Selfe J, Greenhalgh S, Mercer C. A service evaluation of patients suspected of Cauda Equina Syndrome referred to accident and emergency departments from a national telephone triage service. Musculoskelet Sci Pr. 2020/09/13. 2020;50:102248.

15. Hoeritzauer I, Carson A, Statham P, Panicker JN, Granitsiotis V, Eugenicos M, et al. Scan-Negative Cauda Equina Syndrome. Neurology [Internet]. 2021;96(3):e433. Available from: http://n.neurology.org/content/96/3/e433.abstract

16. Hoeritzauer I, Pronin S, Carson A, Statham P, Demetriades AK, Stone J. The clinical features and outcome of scan-negative and scan-positive cases in suspected cauda equina syndrome: a retrospective study of 276 patients. J Neurol. 2018/10/10. 2018;265(12):2916–26.

17. Hogan WB, Kuris EO, Durand WM, Eltorai AEM, Daniels AH. Timing of Surgical Decompression for Cauda Equina Syndrome. World Neurosurg. 2019 Dec;132:e732–8.

18. Hussain MM, Razak AA, Hassan SS, Choudhari KA, Spink GM. Time to implement a national referral pathway for suspected cauda equina syndrome: review and outcome of 250 referrals. Br J Neurosurg. 2018/04/03. 2018;32(3):264–8.

19. Jha V, Deep G, Pandita N, Ahuja K, Ifthekar S, Kandwal P. Factors affecting urinary outcome after delayed decompression in complete cauda equina syndrome: “A regression model study.” Eur J Trauma Emerg Surg. 2021/01/18. 2021;

20. Katzouraki G, Zubairi AJ, Hershkovich O, Grevitt MP. A prospective study of the role of bladder scanning and post-void residual volume measurement in improving diagnostic accuracy of cauda equina syndrome. Bone Joint J [Internet]. 2020;102-B(6):677–82. Available from: https://doi.org/10.1302/0301-620X.102B6.BJJ-2020-0195.R1

21. Kögl N, Brawanski K, Girod PP, Petr O, Thomé C. Early surgery determines recovery of motor deficits in lumbar disc herniations-a prospective single-center study. Acta Neurochir. 2020/11/05. 2021;163(1):275–80.

22. König A, Amelung L, Danne M, Meier U, Lemcke J. Do we know the outcome predictors for cauda equine syndrome (CES)? A retrospective, single-center analysis of 60 patients with CES with a suggestion for a new score to measure severity of symptoms. Eur Spine J. 2017/05/21. 2017;26(10):2565–72.

23. Korse NS, Pijpers JA, van Zwet E, Elzevier HW, Vleggeert-Lankamp CLA. Cauda Equina Syndrome: presentation, outcome, and predictors with focus on micturition, defecation, and sexual dysfunction. Eur Spine J. 2017/01/20. 2017;26(3):894–904.

24. Korse NS, Kruit MC, Peul WC, Vleggeert-Lankamp CLA. Lumbar spinal canal MRI diameter is smaller in herniated disc cauda equina syndrome patients. PLoS One. 2017/10/13. 2017;12(10):e0186148.

25. Korse NS, Veldman AB, Peul WC, Vleggeert-Lankamp CLA. The long term outcome of micturition, defecation and sexual function after spinal surgery for cauda equina syndrome. PLoS One. 2017/04/20. 2017;12(4):e0175987.

26. Krishnan A, Kohli R, Degulmadi D, Mayi S, Ranjan R, Dave B. Cauda Equina Syndrome: A Review of 15 Patients Who Underwent Percutaneous Transforaminal Endoscopic Lumbar Discectomy (PTELD) Under Local Anaesthesia. Malays Orthop J. 2020/09/29. 2020;14(2):101–10.

27. Li X, Dou Q, Hu S, Liu J, Kong Q, Zeng J, et al. Treatment of cauda equina syndrome caused by lumbar disc herniation with percutaneous endoscopic lumbar discectomy. Acta Neurol Belg. 2015/08/22. 2016;116(2):185–90.

28. Pronin S, Hoeritzauer I, Statham PF, Demetriades AK. Are we neglecting sexual function assessment in suspected cauda equina syndrome? Surgeon. 2019/05/01. 2020;18(1):8–11.

29. Savoldi F, Kaufmann TJ, Flanagan EP, Toledano M, Weinshenker BG. Elsberg syndrome: A rarely recognized cause of cauda equina syndrome and lower thoracic myelitis. Neurol Neuroimmunol Neuroinflamm. 2017/05/24. 2017;4(4):e355.

30. Seidel H, Bhattacharjee S, Pirkle S, Shi L, Strelzow J, Lee M, et al. Long-term rates of bladder dysfunction after decompression in patients with cauda equina syndrome. Spine J [Internet]. 2021;21(5):803–9. Available from: https://www.sciencedirect.com/science/article/pii/S1529943021000036

31. Shahmohammadi M, Khoshuod RJ, Zali A, Seddeghi AS, Kabir NM. Examination of The Predictive Power of Electromyography and Urodynamic Study in Patients with Cauda Equina Syndrome (Horse Tail Syndrome). Acta Inform Med [Internet]. 2016/11/01. 2016;24(5):328–31. Available from: https://pubmed.ncbi.nlm.nih.gov/28077887

32. Silva A, Sachdev B, Kostusiak M, Yousif M, Flint G, Dhir J, et al. Out of hours magnetic resonance imaging for suspected cauda equina syndrome: lessons from a comparative study across two centres. Ann R Coll Surg Engl. 2021/03/02. 2021;103(3):218–22.

33. Sath S. Does surgical decompression alleviate neglected cauda equina syndromes attributed to lumbar disc herniation and/or degenerative canal stenosis? Surg Neurol Int [Internet]. 2020;11:278. Available from: https://pubmed.ncbi.nlm.nih.gov/33033640

34. Thakur JD, Storey C, Kalakoti P, Ahmed O, Dossani RH, Menger RP, et al. Early intervention in cauda equina syndrome associated with better outcomes: a myth or reality? Insights from the Nationwide Inpatient Sample database (2005-2011). Spine J. 2017/05/01. 2017;17(10):1435–48.

35. Uçkun ÖM, Alagöz F, Polat Ö, Divanlıoğlu D, Dağlıoğlu E, Belen AD, et al. Urgent operation improves weakness in cauda equina syndrome due to lumbar disc herniation. Turkish J Phys Med Rehabil [Internet]. 2019;65(3):222–7. Available from: https://pubmed.ncbi.nlm.nih.gov/31663070

36. Venkatesan M, Nasto L, Tsegaye M, Grevitt M. Bladder Scans and Postvoid Residual Volume Measurement Improve Diagnostic Accuracy of Cauda Equina Syndrome. Spine (Phila Pa 1976). 2019/09/04. 2019;44(18):1303–8.

37. Woodfield J, Brennan PM, Statham P, Stone J, Hoeritzauer I. Suspected cauda equina syndrome: no reduction in investigation, referral and treatment during the COVID-19 pandemic. Ann R Coll Surg Engl. 2021/03/09. 2021;103(6):432–7.

38. Yang SD, Zhang F, Ding WY. Analysis of clinical and neurological outcomes in patients with cauda equina syndrome caused by acute lumbar disc herniation: a retrospective-prospective study. Oncotarget. 2017/11/16. 2017;8(48):84204–9.

39. Yankang L, Leiming Z, Lewandrowski K-U, Xiangyu T, Zexing Z, Jianbiao X, et al. Full Endoscopic Lumbar Discectomy Versus Laminectomy for Cauda Equina Syndrome. Int J spine Surg [Internet]. 2021/02/12. 2021;15(1):105–12. Available from: https://pubmed.ncbi.nlm.nih.gov/33900963

40. Aly TA, Aboramadan MO. Efficacy of delayed decompression of lumbar disk herniation causing cauda equina syndrome. Orthopedics. 2014/04/01. 2014;37(2):e153-6.

41. Bellabarba C, Schildhauer TA, Vaccaro AR, Chapman JR. Complications associated with surgical stabilization of high-grade sacral fracture dislocations with spino-pelvic instability. Spine (Phila Pa 1976). 2006/05/11. 2006;31(11 Suppl):S80-8; discussion S104.

42. Buchner M, Schiltenwolf M. Cauda equina syndrome caused by intervertebral lumbar disk prolapse: mid-term results of 22 patients and literature review. Orthopedics. 2002/07/26. 2002;25(7):727–31.

43. Busse JW, Bhandari M, Schnittker JB, Reddy K, Dunlop RB. Delayed presentation of cauda equina syndrome secondary to lumbar disc herniation: functional outcomes and health-related quality of life. CJEM. 2007/07/06. 2001;3(4):285–91.

44. Todd N V, Srikandarajah N, Boissaud-Cooke MA, Clark S, Wilby MJ, Shapiro S, et al. Outcome of spinal decompression in Cauda Equina syndrome presenting late in developing countries: case series of 50 cases. Spine (Phila Pa 1976) [Internet]. 2011/05/19. 2011 Dec;25(3):348–52. Available from: https://www.sciencedirect.com/science/article/pii/S0972978X17302672

45. Domen PM, Hofman PA, van Santbrink H, Weber WE. Predictive value of clinical characteristics in patients with suspected cauda equina syndrome. Eur J Neurol. 2009/06/06. 2009;16(3):416–9.

46. Fuso FAF, Dias ALN, Letaif OB, Cristante AF, Marcon RM, de Barros TEP. Epidemiological study of cauda equina syndrome. Acta Ortop Bras [Internet]. 2013;21(3):159–62. Available from: https://pubmed.ncbi.nlm.nih.gov/24453661

47. Galasko CS. Spinal instability secondary to metastatic cancer. J Bone Joint Surg Br [Internet]. 1991;73-B(1):104–8. Available from: https://doi.org/10.1302/0301-620X.73B1.1991739

48. Henriques T, Olerud C, Petrén-Mallmin M, Ahl T. Cauda equina syndrome as a postoperative complication in five patients operated for lumbar disc herniation. Spine (Phila Pa 1976). 2001/02/27. 2001;26(3):293–7.

49. Hussain SA, Gullan RW, Chitnavis BP. Cauda equina syndrome: outcome and implications for management. Br J Neurosurg. 2003/06/25. 2003;17(2):164–7.

50. McCarthy MJ, Aylott CE, Grevitt MP, Hegarty J. Cauda equina syndrome: factors affecting long-term functional and sphincteric outcome. Spine (Phila Pa 1976). 2007/01/17. 2007;32(2):207–16.

51. Kennedy JG, Soffe KE, McGrath A, Stephens MM, Walsh MG, McManus F. Predictors of outcome in cauda equina syndrome. Eur Spine J. 1999/09/14. 1999;8(4):317–22.

52. Ng LCL, Tafazal S, Longworth S, Sell P. Cauda equina syndrome: an audit. Can we do better. 2004;98–101.

53. Olivero WC, Wang H, Hanigan WC, Henderson JP, Tracy PT, Elwood PW, et al. Cauda equina syndrome (CES) from lumbar disc herniations. J Spinal Disord Tech. 2009/05/05. 2009;22(3):202–6.

54. Qureshi A, Sell P. Cauda equina syndrome treated by surgical decompression: the influence of timing on surgical outcome. Eur Spine J. 2007/09/11. 2007;16(12):2143–51.

55. Raj D, Coleman N. Cauda equina syndrome secondary to lumbar disc herniation. Acta Orthop Belg. 2008/09/25. 2008;74(4):522–7.

56. Sengoz A, Kotil K, Tasdemiroglu E. Posterior epidural migration of herniated lumbar disc fragment. J Neurosurg Spine. 2011/01/22. 2011;14(3):313–7.

57. Shapiro S. Cauda equina syndrome secondary to lumbar disc herniation. Neurosurgery. 1993/05/01. 1993;32(5):743–7.

58. Shapiro S. Medical realities of cauda equina syndrome secondary to lumbar disc herniation. Spine (Phila Pa 1976). 2000;25(3):348–52.

59. Shen L, Fang L, Qiu Y, Xing S, Chen D, He X, et al. Study on different surgical approaches for acute Lumber disk protrusion combined with Cauda Equina Syndrome. Int J Clin Exp Pathol [Internet]. 2014;7(12):8875–80. Available from: https://pubmed.ncbi.nlm.nih.gov/25674258

60. Sokolowski MJ, Garvey TA, Perl 2nd J, Sokolowski MS, Akesen B, Mehbod AA, et al. Postoperative lumbar epidural hematoma: does size really matter? Spine (Phila Pa 1976). 2008/01/01. 2008;33(1):114–9.

61. Srikandarajah N, Boissaud-Cooke MA, Clark S, Wilby MJ. Does early surgical decompression in cauda equina syndrome improve bladder outcome? Spine (Phila Pa 1976). 2015 Apr;40(8):580–3.

62. Szövérfi Z, Lazary A, Bozsódi Á, Klemencsics I, Éltes PE, Varga PP. Primary Spinal Tumor Mortality Score (PSTMS): a novel scoring system for predicting poor survival. Spine J. 2014/03/22. 2014;14(11):2691–700.

63. Tamburrelli FC, Genitiempo M, Bochicchio M, Donisi L, Ratto C. Cauda equina syndrome: evaluation of the clinical outcome. Eur Rev Med Pharmacol Sci. 2014/04/26. 2014;18(7):1098–105.

64. Todd N V. Causes and outcomes of cauda equina syndrome in medico-legal practice: a single neurosurgical experience of 40 consecutive cases. Br J Neurosurg. 2011 Aug;25(4):503–8.

65. Wostrack M, Shiban E, Obermueller T, Gempt J, Meyer B, Ringel F. Conus medullaris and cauda equina tumors: clinical presentation, prognosis, and outcome after surgical treatment: clinical article. J Neurosurg Spine. 2014/01/21. 2014;20(3):335–43.

66. Yamanishi T, Yasuda K, Yuki T, Sakakibara R, Uchiyama T, Kamai T, et al. Urodynamic evaluation of surgical outcome in patients with urinary retention due to central lumbar disc prolapse. Neurourol Urodyn. 2003/11/05. 2003;22(7):670–5.

67. Galvin JW, Freedman BA, Schoenfeld AJ, Cap AP, Mok JM. Morbidity of early spine surgery in the multiply injured patient. Arch Orthop Trauma Surg. 2014/08/01. 2014;134(9):1211–7.

68. Sapkas GS, Mavrogenis AF, Papagelopoulos PJ. Transverse sacral fractures with anterior displacement. Eur Spine J [Internet]. 2007/11/14. 2008;17(3):342–7. Available from: https://pubmed.ncbi.nlm.nih.gov/18000690

69. Schildhauer TA, Bellabarba C, Nork SE, Barei DP, Routt Jr. ML, Chapman JR. Decompression and lumbopelvic fixation for sacral fracture-dislocations with spino-pelvic dissociation. J Orthop Trauma. 2006/08/08. 2006;20(7):447–57.

70. Sun T, Liu Z, Liu S, Xu S. The clinical study of repairing cauda equina fibres with fibrin glue after lumbar fracture and dislocation. Spinal Cord [Internet]. 2010;48(8):633–7. Available from: https://doi.org/10.1038/sc.2009.195

71. Tan GQ, He JL, Fu BS, Li LX, Wang BM, Zhou DS. Lumbopelvic fixation for multiplanar sacral fractures with spinopelvic instability. Injury. 2012/05/29. 2012;43(8):1318–25.

72. Arrigo RT, Kalanithi P, Boakye M. Is cauda equina syndrome being treated within the recommended time frame? Neurosurgery. 2011/02/12. 2011;68(6):1520–6; discussion 1526.

73. Kotil K, Eras M, Akçetin M, Acar C, Bilge T. Do the spinal pathologies that accompany lumbar disc disease affect surgical prognosis? Turk Neurosurg. 2006;16:168–74.

74. Shi J, Jia L, Yuan W, Shi G, Ma B, Wang B, et al. Clinical classification of cauda equina syndrome for proper treatment. Acta Orthop. 2010 Jun;81(3):391–5.

75. Akbar A, Mahar A. Lumbar disc prolapse: management and outcome analysis of 96 surgically treated patients. J Pak Med Assoc. 2002/06/21. 2002;52(2):62–5.

76. Allegretti L, Mavilio N, Fiaschi P, Bragazzi R, Pacetti M, Castelletti L, et al. Intra-operative vertebroplasty combined with posterior cord decompression. A report of twelve cases. Interv Neuroradiol [Internet]. 2014/10/17. 2014;20(5):583–90. Available from: https://pubmed.ncbi.nlm.nih.gov/25363261

77. Ayoub MA. Displaced spinopelvic dissociation with sacral cauda equina syndrome: outcome of surgical decompression with a preliminary management algorithm. Eur Spine J [Internet]. 2012/06/26. 2012;21(9):1815–25. Available from: https://pubmed.ncbi.nlm.nih.gov/22732828

78. Baba H, Maezawa Y, Furusawa N, Imura S, Tomita K. The role of calcium deposition in the ligamentum flavum causing a cauda equina syndrome and lumbar radiculopathy. Spinal Cord [Internet]. 1995;33(4):219–23. Available from: https://doi.org/10.1038/sc.1995.49

79. Bejia I, Younes M, Zrour S, Touzi M, Bergaoui N. Factors predicting outcomes of mechanical sciatica: a review of 1092 cases. Jt Bone Spine. 2004/12/14. 2004;71(6):567–71.

80. Božić B, Kogler A, Negovetić L, Sajko T, Kudelić N. Sequestered extrusion of lumbar disk: Experimental model, clinical picture, diagnosis and treatment. Acta Clin Croat. 2003;42(3):213–6.

81. Crocker M, Fraser G, Boyd E, Wilson J, Chitnavis BP, Thomas NW. The value of interhospital transfer and emergency MRI for suspected cauda equina syndrome: a 2-year retrospective study. Ann R Coll Surg Engl. 2008/07/05. 2008;90(6):513–6.

82. Duncan JW, Bailey RA. Cauda equina syndrome following decompression for spinal stenosis. Glob spine J [Internet]. 2011;1(1):15–8. Available from: https://pubmed.ncbi.nlm.nih.gov/24353932

83. Ea HK, Lioté F, Lot G, Bardin T. Cauda equina syndrome in ankylosing spondylitis: successful treatment with lumboperitoneal shunting. Spine (Phila Pa 1976). 2010/10/30. 2010;35(24):E1423-9.

84. Fukui J, Ohotsuka K, Asagai Y. Improved symptoms and lifestyle more than 20 years after untethering surgery for primary tethered cord syndrome. Neurourol Urodyn. 2011/06/01. 2011;30(7):1333–7.

85. Gooding BW, Higgins MA, Calthorpe DA. Does rectal examination have any value in the clinical diagnosis of cauda equina syndrome? Br J Neurosurg. 2012/11/02. 2013;27(2):156–9.

86. Lyons MK, Atkinson JL, Wharen RE, Deen HG, Zimmerman RS, Lemens SM. Surgical evaluation and management of lumbar synovial cysts: the Mayo Clinic experience. J Neurosurg. 2000/07/06. 2000;93(1 Suppl):53–7.

87. Marascalchi BJ, Passias PG, Goz V, Weinreb JH, Joo L, Errico TJ. Comparative analysis of patients with cauda equina syndrome versus an unaffected population undergoing spinal surgery. Spine (Phila Pa 1976). 2013/12/25. 2014;39(6):482–90.

88. McKinley WO, Tellis AA, Cifu DX, Johnson MA, Kubal WS, Keyser-Marcus L, et al. Rehabilitation outcome of individuals with nontraumatic myelopathy resulting from spinal stenosis. J Spinal Cord Med. 1998/08/11. 1998;21(2):131–6.

89. Morita M, Miyauchi A, Okuda S, Oda T, Aono H, Iwasaki M. Intraspinal epidermoid tumor of the cauda equina region: seven cases and a review of the literature. J Spinal Disord Tech. 2011/05/24. 2012;25(5):292–8.

90. Ökten AI, Özsoy KM, Gezercan Y, Ateş T, Menekşe G, Çapraz M, et al. Analysis of clinical and surgical outcomes of upper lumbar disk herniations. Neurosurg Q. 2015;25(3):349–54.

91. Podnar S. Cauda equina lesions as a complication of spinal surgery. Eur Spine J [Internet]. 2009/09/21. 2010;19(3):451–7. Available from: https://pubmed.ncbi.nlm.nih.gov/19768646

92. Ronen J, Goldin D, Itzkovich M, Bluvshtein V, Gelernter I, Livshitz A, et al. Outcomes in patients admitted for rehabilitation with spinal cord or cauda equina lesions following degenerative spinal stenosis. Disabil Rehabil. 2005/08/13. 2005;27(15):884–9.

93. Sakai Y, Matsuyama Y, Katayama Y, Imagama S, Ito Z, Wakao N, et al. Spinal myxopapillary ependymoma: neurological deterioration in patients treated with surgery. Spine (Phila Pa 1976). 2009/07/01. 2009;34(15):1619–24.

94. Smith MD, Bohlman HH. Spondylolisthesis treated by a single-stage operation combining decompression with in situ posterolateral and anterior fusion. An analysis of eleven patients who had long-term follow-up. J Bone Jt Surg Am. 1990/03/01. 1990;72(3):415–21.

95. Takahashi T, Hanakita J, Kawaoka T, Ohtake Y, Adachi H, Shimizu K. Indication for Partial Vertebral Osteotomy and Realignment in Posterior Spinal Fixation for Osteoporotic Thoracolumbar Vertebral Collapse with Neurological Deficits. Neurol Med Chir. 2016/03/30. 2016;56(8):485–92.

96. Walker JL, Schulak D, Murtagh R. Midline disk herniations of the lumbar spine. South Med J. 1993/01/01. 1993;86(1):13–7.

97. Baig Mirza A, Velicu MA, Lyon R, Vastani A, Boardman T, Al Banna Q, et al. Is Cauda Equina Surgery Safe Out-of-Hours? A Single United Kingdom Institute Experience. World Neurosurg [Internet]. 2022;159:e208–20. Available from: https://www.sciencedirect.com/science/article/pii/S1878875021018714

98. Baraka M, Varma A, Mayo I, Nannapaneni R, McGillion S, Shenouda E, et al. The Effect of COVID-19 National Lockdown on the Time from Presentation to Surgery of Patients with Suspected Cauda Equina Syndrome: Two UK Tertiary Centers’ Study. World Neurosurg. 2022 Aug;167:e732-7.

99. Butenschoen VM, Abulhala S, Meyer B, Gempt J. Body mass index has an impact on preoperative symptoms but not clinical outcome in acute cauda equina syndrome. Sci Rep [Internet]. 2021;11(1):13918. Available from: https://doi.org/10.1038/s41598-021-92969-4

100. Byvaltsev VA, Kalinin AA, Shepelev V V, Pestryakov YY, Aliyev MA, Riew KD. Minimally Invasive Transforaminal Lumbar Interbody Fusion (TLIF) Compared with Open TLIF for Acute Cauda Equina Syndrome: A Retrospective Single-Center Study with Long-Term Follow-Up. World Neurosurg. 2022 Oct;166:e781–9.

101. Curtis Lopez C, Berg AJ, Clayton B, Siddique I, Carrasco R, Horner D, et al. Evaluation of the role of anal tone and perianal sensation examination in the assessment of suspected cauda equina syndrome. Br J Neurosurg. 2021 Nov;1–5.

102. Francis JJ, Goacher E, Fuge J, Hanrahan JG, Zhang J, Davies B, et al. Lumbar decompression surgery for cauda equina syndrome — comparison of complication rates between daytime and overnight operating. Acta Neurochir (Wien) [Internet]. 2022;164(5):1203–8. Available from: https://doi.org/10.1007/s00701-022-05173-2

103. Kanematsu R, Hanakita J, Takahashi T, Minami M, Inoue T, Miyasaka K, et al. Improvement in Neurogenic Bowel and Bladder Dysfunction Following Posterior Decompression Surgery for Cauda Equina Syndrome: A Prospective Cohort Study. Neurospine. 2021 Dec;18(4):847–53.

104. Bhandarkar AR, Alvi MA, Naessens JM, Bydon M. Do safety-net hospitals provide equitable care after decompressive surgery for acute cauda equina syndrome? Clin Neurol Neurosurg. 2021 Jan;200:106356.

105. Woodfield J, Lammy S, Jamjoom AA, Fadelalla MA, Copley PC, Arora M, et al. Demographics of Cauda Equina Syndrome: A Population Based Incidence Study. Neuroepidemiology. 2022 Oct;

106. Zusman NL, Radoslovich SS, Smith SJ, Tanski M, Gundle KR, Yoo JU. Physical Examination Is Predictive of Cauda Equina Syndrome: MRI to Rule Out Diagnosis Is Unnecessary. Glob spine J. 2022 Mar;12(2):209–14.

107. Todd N, Dangas K, Lavy C. Post-void bladder ultrasound in suspected cauda equina syndrome-data from medicolegal cases and relevance to magnetic resonance imaging scanning. Int Orthop. 2022 Jun;46(6):1375–80.

108. Kumar A, Copley P, Jamjoom A, Badran K, Barrett C. The morbidity of out-of-hours surgery for Cauda Equina Syndrome. Br J Neurosurg. 2022 Jan;1–4.

109. Seidel HD, Pirkle S, Bhattacharjee S, Baker HP, Lee MJ, El Dafrawy MH. Rates of Future Lumbar Fusion in Patients with Cauda Equina Syndrome Treated With Decompression. J Am Acad Orthop Surg Glob Res Rev. 2022 Nov;6(11).

110. Planty-Bonjour A, Kerdiles G, François P, Destrieux C, Velut S, Zemmoura I, et al. Cauda Equina Syndrome: Poor Recovery Prognosis Despite Early Treatment. Spine (Phila Pa 1976). 2022 Jan;47(2):105–13.
