# Supplementary material for: Definition and surgical timing in cauda equina syndrome–An updated systematic review
Source: PLoS One. 2023 May 4;18(5):e0285006. doi: 10.1371/journal.pone.0285006 (PMC10159340; doi:10.1371/journal.pone.0285006)
Supplement: Attachment — Submitted filename: Response to Reviewers.docx [file pone.0285006.s004.docx]

Conor S. Gillespie

Institute of Systems, Molecular and Integrative Biology

University of Liverpool

Liverpool

L69 7BE, UK

21 July 2022

Dr. Alfio Spina	Academic Editor,	PLOS One

Dear Alfio Spina,

**Re: Definition and surgical timing in Cauda Equina Syndrome – A systematic review**

Thank you considering our revised manuscript entitled “Definition and surgical timing in Cauda Equina Syndrome – A systematic review” for publication in PLOS One. We thank the reviewers for their constructive comments and feedback. We hope we have addressed their concerns adequately in our revision of the manuscript and attach our point-by-point responses below. We believe that this manuscript is an important addition to the literature and the revisions have strongly enhanced this message.

I would like to confirm again that with this submission, all study authors declare that they have reviewed the final manuscript, approved the contents of it and that the requirements for authorship have been met by all named authors. We can guarantee that the work is not being considered for publication by any other journal and that no previous work we’ve published (apart from meeting abstracts) overlap with the current work. We have not received any external funding for the completion of this work and have no relevant conflicts of interest to report.

I thank you once again for considering our manuscript for publication and look forward to hearing from you soon.

Yours Sincerely,

Conor S. Gillespie	MPhil student	Institute of Systems, Molecular and Integrative Biology (ISMIB)	University of Liverpool, UK	Email: [hlcgill2@liv.ac.uk](mailto:hlcgill2@liv.ac.uk)

**Response to Associate Editor comments:**

1. Please ensure that your manuscript meets PLOS ONE’s style requirements, including those for file naming.

*A: Thank you for highlighting this discrepancy and providing the link to the author and affiliation style format. We have now edited the title page in line with PLOS ONE’s style requirements.*

2. Please provide additional details regarding participant consent. In the ethics statement in the Methods and online submission information, please ensure that you have specified (1) whether consent as informed and (2) what type you obtained (for instance, written or verbal, how it was documented and witnessed). If your study included minors, state whether you obtained consent from parents or guardians. If the need for consent was waived by the ethics committee, please include this information.

If you are reporting a retrospective study of medical records or archived samples, please ensure that you have discussed whether all data were fully anonymized before you accessed them and/or whether the IRB or ethics committee waived the requirement for informed consent. If patients provided informed written consent to have data from their medical records used in research, please include this information.

*A: Thank you for highlighting this point. As this is a systematic review including published studies and presenting data at a study level, individual participant consent was not required. We have specified the same in the ethics statement in the Methods (Lines 156-159, Revised Manuscript with Track Changes) and the online submission information field.*

3. Thank you for stating the following in the Funding Section of your manuscript: “The authors did not…grant from the Wolfson Foundation”.

We note that you have provided funding information that is not currently declared in your Funding Statement. However, funding information should not appear in the Acknowledgements section or other areas of your manuscript. We will only publish funding information present in the Funding Statement section of the online submission form. Please remove any funding-related text from the manuscript and let us know how you would like to update your Funding Statement. Currently, your Funding Statement reads as follows: “The author(s) received no specific funding for this work”.

*A: Thank you for highlighting this discrepancy between the Funding Section of our manuscript and the Funding Statement. For consistency we have removed the funding section from our manuscript. We would like the Funding Statement to read as “The author(s) received no specific funding to complete this work. The manuscript publication costs are funded by the University of Liverpool”.*

4. We note that you have indicated that data from this study are available upon request. PLOS only allows data to ab. We note that you have indicated that data from this study are available upon request. PLOS only allows data to be available upon request if there are legal or ethical restrictions on sharing data publicly. For more information on unacceptable data access restrictions, please see http://journals.plos.org/plosone/s/data-availability#loc-unacceptable-data-access-restrictions.

In your revised cover letter, please address the following prompts:

a) If there are ethical or legal restrictions on sharing a de-identified data set, please explain them in detail (e.g., data contain potentially sensitive information, data are owned by a third-party organization, etc.) and who has imposed them (e.g., an ethics committee). Please also provide contact information for a data access committee, ethics committee, or other institutional body to which data requests may be sent.

b) If there are no restrictions, please upload the minimal anonymized data set necessary to replicate your study findings as either Supporting Information files or to a stable, public repository and provide us with the relevant URLs, DOIs, or accession numbers. For a list of acceptable repositories, please see http://journals.plos.org/plosone/s/data-availability#loc-recommended-repositories.

We will update your Data Availability statement on your behalf to reflect the information you provide.

*A: a) We confirm that there are no ethical or legal restrictions on sharing our dataset.*

*(b) Alongside the revised manuscript, we will upload a minimal anonymized data set containing data points used in our analysis. The dataset will be uploaded as a Microsoft Excel file.*

5. Review Comments to the Author

Please use the space provided to explain your answers to the questions above. You may also include additional comments for the author, including concerns about dual publication, research ethics, or publication ethics. (Please upload your review as an attachment if it exceeds 20,000 characters)

Reviewer #1: This is an excellent review of a timely topic. If the same language and definition of time is not used then it is impossible to determine the results across studies. This work will highlight that need.

Reviewer #2: This is a very interesting study concerning the definition and surgical timing in cauda equinal syndrome. But the varieties of the syndrome were highly correlated to the etiologies of disease. The surgical timing also depended on the diseases themselves.
The authors should add the parameters of etiologies as a crucial factor for the analysis.

*A: Reviewer #1 – Thank you very much for reviewing our paper and the positive comment. We wholeheartedly agree with this and recognise the need for establishing a consensus for CES being a relevant topic.*

*Reviewer #2 – Thank you very much for reviewing our paper and highlighting the importance of CES etiologies in our analysis. We have now included a section with descriptive statistics outlining the etiologies in included papers in our Results section (Line 174-178, Revised Manuscript with Track Changes).*
