# Supplementary material for: Definition and surgical timing in cauda equina syndrome–An updated systematic review
Source: PLoS One. 2023 May 4;18(5):e0285006. doi: 10.1371/journal.pone.0285006 (PMC10159340; doi:10.1371/journal.pone.0285006)
Supplement: Attachment — Submitted filename: Response to Reviewers.docx [file pone.0285006.s005.docx]

Conor S. Gillespie

Institute of Systems, Molecular and Integrative Biology

University of Liverpool

Liverpool

L69 7BE, UK

29^th^ January 2023

Dr. Andreas K. Demetriades,	Academic Editor,	PLOS One

Dear Dr. Demetriades,

**Re: Definition and surgical timing in Cauda Equina Syndrome – A systematic review**

Thank you considering our revised manuscript entitled “Definition and surgical timing in Cauda Equina Syndrome – A systematic review” for publication in PLOS One. We thank the reviewers for their constructive comments and feedback. We hope we have addressed their concerns adequately in our revision of the manuscript and attach our point-by-point responses below. We believe that this manuscript is an important addition to the literature and the revisions have strongly enhanced this message.

I would like to confirm again that with this submission, all study authors declare that they have reviewed the final manuscript, approved the contents of it and that the requirements for authorship have been met by all named authors. We can guarantee that the work is not being considered for publication by any other journal and that no previous work we’ve published (apart from meeting abstracts) overlap with the current work. We have not received any external funding for the completion of this work and have no relevant conflicts of interest to report.

I thank you once again for considering our manuscript for publication and look forward to hearing from you soon.

Yours Sincerely,

Conor S. Gillespie	Honorary Clinical Associate	Institute of Systems, Molecular and Integrative Biology (ISMIB)	University of Liverpool, UK	Email: [hlcgill2@liv.ac.uk](mailto:hlcgill2@liv.ac.uk)

**Response to review comments to the author:**

**Reviewer #1:** The authors present an acceptable version of their paper. The authors present an acceptable version of their paper.

**Reviewer #2:** The authors had already addressed all the response to comments recommended by the reviewer. The manuscript should be published.

*A: Reviewer #1 – Thank you very much for reviewing our paper and the positive comment. We wholeheartedly agree with this and recognise the need for establishing a consensus for CES being a relevant topic.*

**Reviewer #3:**

1. Title: I suggest that the title be revised to illustrate that this is an “updated” systematic review

*A: Thank you for highlighting this point. We have now added “updated” to our title to reflect our choice to build on the systematic review published by Mr. Nisaharan Srikandarajah, titled, “Outcomes Reported After Surgery for Cauda Equina Syndrome” (2018, Spine), as it followed the same inclusion criteria to our review.*

2. Abstract:

a) Study design: narrative review -> please revise

*A: Thank you for highlighting this. We have now changed the Study Design section in the Abstract to “Systematic review”.*

b) Be consistent in your use of “n” for study number and the use of percentages

*A: For consistency we have now used the total study number as “n” throughout this section and changed the percentages to reflect the same.*

3. Methods:

a) Search strategy: The literature search is outdated (last update: 30th April 2021). Please re-run the literature search to identify articles published in the last 1.5 years.

*A: We have now updated the systematic review and included all articles identified as per our last search (30^th^ December 2022). We have now added a further 14 articles to our results.*

b) Search strategy: Although the PRISMA guidelines only require the strategy of at least 1 database to be presented, I suggest that the search strategy for all databases is presented in Table S1.

*A: Thank you for the above suggestion. We have now added the search strategy for all three databases to the electronic supplementary material.*

c) Study screening and selection: Lines 123-126, please revise the sentence.

*A: Thank you for highlighting the above. We have now re-structured lines 123-126, Methods, for clarity. The section now reads as follows: “*After removal of duplicates, titles and abstracts were screened against the population, intervention, comparison, outcome, and study design (PICOS) criteria, where outcome referred to study characteristic, defined in Table 1 by two independent, blinded reviewers (GER and CSG). Following this, full-texts were screened by two independent, blinded reviewers (MAM and GER), to confirm manuscripts eligible for inclusion. All manuscripts eligible for inclusion were cross-checked by the senior author (NS).”

d) Statistical analysis: please mention the statistical tests that yielded the p values mentioned later in the text

*A: Thank you for highlighting the lack of an appropriate statistical mentioned. We have now stated that categorical variables were compared using the Chi-Squared test (Line 153, Results, Statistical Analysis).*

e) Quality assessment: please provide a quality assessment paragraph as mentioned in your PRISMA protocol. A paragraph with the results should also be provided in the "Results" section.

*A: Thank you for highlighting the above. We decided against conducting risk of bias and quality assessments for this review as we did not conduct any synthetic analysis and only used descriptive methods to describe the heterogeneity in CES definition and time to surgery details. We included all studies that matched our inclusion criteria due to the heterogenous literature consisting of predominantly retrospective studies. To reflect the above we have now submitted an amendment to our PROSPERO form.*

4. Results:

a) I believe that combining some of the data of the supplementary tables to create 1-2 Tables presented in the main text with the basic characteristics of the included studies will help readers better understand the findings. (suggestion)

*A: Thank you for the above suggestion. We have now combined some of the data in the supplementary tables and incorporated Table S4 (previously S2), into the main text of the manuscript. We hope this adds to the manuscript by providing more information about the included papers to the readers.*

b) Be consistent in your use of "n" for study number and the use of percentages

*A: Thank you pointing out the above. We have used the total number of studies as the “n” throughout the results section for clarity.*

c) Please provide statistical significance values when comparing the two periods (1990-2016 & 2016-2021). For example here: "There was an increase in percentage of studies using pre-existing criteria over the time period
205 2016-2021 compared to the ones published in 1990-2016, with 9.4% of studies using criteria 206 between 1990-2016 (n=3), and 38.7% (n=12) of studies using them between the years 2016-207 2021."

*A: We have now added p-values from the Chi-Squared test conducted to compare the categorical variables. We hope this emphasises the difference in the above rates of studies defining CES and reporting time to surgery.*

5. Discussion:

a) Make sure to include references consistently when mentioning other studies

*A: Thank you for highlighting the above. We have revised the discussion section as per comments from all reviewers and included consistent references throughout.*

**Reviewer #4:** Authors present a revised version of the narrative review of definiton and surgical timing in cauda equina syndrome. Methodology of study selection is fairly well described. Authors conclude that there is currently no concensus , either on definiton nor on how early is the early timing in management of cauda equina syndrome.

One major drawback of this study is that it does not discuss one very important aspect of the studies which were analyzed - the etiology. Cauda equina syndrome can appear due to tumor compression - primary or metastasis, due to trauma, infection or degenerative disease. We are sure that there would be certain differences in the management, timing and surgical treatment in general; what authors did in the revision is just information of which etiologies in which percentage led to the syndrome. Furthermore, this review provides a mere description of the evaluated studies, without any comparison between the studies; surgical philosophy is not discussed at all (decompression surgery, decompression + stabilization), and one very important clinical aspect - not only time of the surgery in terms of from onset of symptoms to surgery, but also time of the day of the surgery - were there any studies which analyze this very important aspect and its correlation to timing? - for example:

Baig Mirza A, Velicu MA, Lyon R, Vastani A, Boardman T, Al Banna Q, Murphy C, Kellett C, Vasan AK, Grahovac G. Is Cauda Equina Surgery Safe Out-of-Hours? A Single United Kingdom Institute Experience. World Neurosurg. 2022 Mar;159:e208-e220. doi: 10.1016/j.wneu.2021.12.028. Epub 2021 Dec 14. PMID: 34915208.

Demetriades AK. Cauda equina syndrome - from timely treatment to the timing of out-of-hours surgery. Acta Neurochir (Wien). 2022 May;164(5):1201-1202. doi: 10.1007/s00701-022-05174-1. Epub 2022 Mar 30. PMID: 35352153.

Are there any information of complication rates of these surgeries? To simply describe timing without description of outcome does not make a lot of sense, so I suggest to include outcome assesment following surgery in correlation to time, and to include into Discussion and comment on current literature:

Woodfield J, Lammy S, Jamjoom AA, Fadelalla MA, Copley PC, Arora M, Glasmacher SA, Abdelsadg M, Scicluna G, Poon MT, Pronin S, Leung AH, Darwish S, Demetriades AK, Brown J, Eames N, Statham PF, Hoeritzauer I; UCES Study Collaborators; British Neurosurgical Trainee Research Collaborative. Demographics of Cauda Equina Syndrome: A Population Based Incidence Study. Neuroepidemiology. 2022 Oct 31. doi: 10.1159/000527727. Epub ahead of print. PMID: 36315989.

I urge authors to analyze the following manuscript and compare their results to this review:
Epstein NE. Review/Perspective: Operations for Cauda Equina Syndromes - "The Sooner the Better". Surg Neurol Int. 2022 Mar 25;13:100. doi: 10.25259/SNI_170_2022. PMID: 35399881; PMCID: PMC8986648.

*A: Thank you for reviewing our manuscript and providing the above comments. Following the updated search, we have now included the above three articles in our review. The scope of this study was to address the definition of CES and how timing of CES is reported. Aetiology and surgical procedures undertaken were not discussed considering volume of content reported and readability. Thank you for highlighting this as it is an area, we can focus our future work on. We have now added to the role of time of surgery in the discussion. We hope this provides readers with more information about CES and adds to the evidence regarding the heterogeneity of the condition and the current lack of consensus with its definition. This should further emphasise the need for a consistent definition of CES and agreement for the time-point at which time to surgery should be measured from.*
