# Supplementary material for: Definition and surgical timing in cauda equina syndrome–An updated systematic review
Source: PLoS One. 2023 May 4;18(5):e0285006. doi: 10.1371/journal.pone.0285006 (PMC10159340; doi:10.1371/journal.pone.0285006)
Supplement: Attachment — Submitted filename: Response to Reviewers (1).docx [file pone.0285006.s006.docx]

Conor S. Gillespie

Institute of Systems, Molecular and Integrative Biology

University of Liverpool

Liverpool

L69 7BE, UK

2^nd^ April 2023

Dr. Andreas K. Demetriades,	Academic Editor,	PLOS One

Dear Dr. Demetriades,

**Re: Definition and surgical timing in Cauda Equina Syndrome – An updated systematic review**

Thank you considering our revised manuscript entitled “Definition and surgical timing in Cauda Equina Syndrome – A systematic review” for publication in PLOS One. We thank the reviewers for their constructive comments and feedback. We hope we have addressed their concerns adequately in our revision of the manuscript and attach our point-by-point responses below. We believe that this manuscript is an important addition to the literature and the revisions have strongly enhanced this message.

I would like to confirm again that with this submission, all study authors declare that they have reviewed the final manuscript, approved the contents of it and that the requirements for authorship have been met by all named authors. We can guarantee that the work is not being considered for publication by any other journal and that no previous work we’ve published (apart from meeting abstracts) overlap with the current work. We have not received any external funding for the completion of this work and have no relevant conflicts of interest to report.

I thank you once again for considering our manuscript for publication and look forward to hearing from you soon.

Yours Sincerely,

Conor S. Gillespie	Honorary Clinical Associate	Institute of Systems, Molecular and Integrative Biology (ISMIB)	University of Liverpool, UK	Email: [hlcgill2@liv.ac.uk](mailto:hlcgill2@liv.ac.uk)

**Response to review comments to the author:**

**Reviewer #4:** Authors have provided a revised version of their manuscript on timing of surgery in cases of cauda equine syndrome. Recommended literature has been included and discussed. Authors state that due to the volume of the analyzed studies, further inquiry regarding etiology which led to cauda syndrome as well as the role of surgical therapy could not have been analyzed. I urge authors to go through the studies and look at this issue, even more data of descriptive nature could be of interest and enrich the manuscript. Especially timing in relation to trauma and tumors has a different significance and dynamics.

*A: Thank you for raising the above point regarding addition of the CES etiology and role of surgical therapy to our manuscript. We now have a descriptive section outlining the underlying etiology of CES (Results, Line 171-175). We have also included a section in our results, titled “Surgical Method”, Line 217-229, describing the predominant surgical method employed for studies included. In addition, our discussion (Line 275-279) now acknowledges the role of CES etiology in the heterogeneity of this condition. We hope these changes have strengthened our review and provided readers with relevant information regarding the CES etiology and surgical methods reported in the literature.*
